# The Hidden Crux of Correctly Determining Octanol–Water Partition Coefficients

**DOI:** 10.1021/acs.molpharmaceut.5c00552

**Published:** 2025-07-03

**Authors:** Espen Fritschka, Gabriele Sadowski

**Affiliations:** † TU Dortmund University, Department of Biochemical and Chemical Engineering, Laboratory of Thermodynamics, Emil-Figge-Straße 70, D-44227 Dortmund, Germany; ‡ Research Center Chemical Sciences and Sustainability, Research Alliance Ruhr, D-44780 Bochum, Germany

**Keywords:** *K*_OW_, log* P*, partition coefficient, distribution coefficient, solubility ratio, extrapolation, ionization

## Abstract

The partitioning of molecules between an aqueous and an organic medium is of major interest for pharmaceutical development and the chemical industry. It characterizes the impact of substances to the environment and to humans, e.g., their accumulation in living organisms. It is usually quantified in terms of the octanol–water partition coefficient *K*_OW_ of these substances. Although this is a clearly defined thermodynamic property, different experimental approaches exist for its estimation. Using active pharmaceutical ingredients (APIs) as examples, we demonstrate the large scatter in experimentally determined partition coefficients reported in the literature. This is especially serious for weak bases or weak acids, which account for around 95% of all APIs. In some cases, reported *K*_OW_ values for the same substance differ by even several orders of magnitude. This is particularly worrying because this property is crucial for approval procedures of APIs and is also used as input for a whole range of estimation methods, such as machine-learning algorithms. In this work, we discuss the physical reasons for the unusually high variety of reported *K*_OW_ values. Using physicochemical laws, it is shown that the large scatter of the data is not caused by analytical uncertainties but by the extrapolation of the experimental data to a solute concentration of zero. Based on this, we propose a new approach for evaluating experimental data on partition coefficients. This approach involves extrapolating experimentally determined distribution coefficients with respect to pH rather than concentration. We will show that this reduces the uncertainty of the experimentally obtained *K*_OW_ values, narrowing the difference between the highest and the lowest value for the same substance of currently about 2.4 to about 0.5 logarithmic units. The new approach can be combined with any existing experimental method for concentration analysis. Moreover, the obtained data agree very well with theoretical values obtained from thermodynamic modeling explicitly considering solute ionization, thus validating the proposed approach.

## Graphical abstract

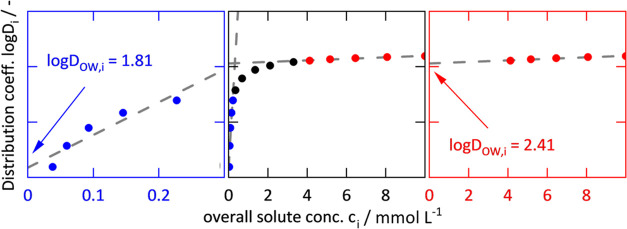

## 1 Introduction

The hydrophilicity or lipophilicity of a substance is often indicated by the octanol–water partition coefficient, the so-called *K*_OW_ value. This property describes whether a substance is preferably found in the octanol-rich phase or in the aqueous phase when present in a biphasic mixture of octanol and water. *K*_OW_ values higher than 1 mean that the majority of the substance is distributed into the organic phase, and therefore the substance is lipophilic.^1–3^ Otherwise, it is hydrophilic.

The *K*_OW_ value is often used to estimate the accumulation of a substance in an organic environment as it is important to ensure that the residues of hazardous substances do not accumulate in nature and poison organisms.^4^

One of the most relevant application areas of *K*_OW_ values is pharmaceutics.^5,6^ To approve a new active pharmaceutical ingredient (API), its *K*_OW_ value must be specified. However, although organization for economic co-operation and development (OECD) guidelines 107 ^7^ and 117 ^8^ provide standardized regulations for determining *K*_OW_ values, many different methods are commonly used in practice. This results in a wide range of reported *K*_OW_ values for the same substance.^9^ The deviations in reported values exceed experimental uncertainties by far, even by multiple orders of magnitude. Several publications have already drawn attention to this discrepancy. Of particular note is a paper series from the Avdeef group, which discusses a wide variety of substance classes and use cases in great detail.^10,11^

Moreover, various theoretical approaches exist to predict *K*_OW_ values.^12–15^ The most widely used tools are quantitative structure–activity relationship (QSAR) and Xlog*P*3. More recently, machine-learning algorithms are used for this purpose.^16–18^ They rely on large data sets, whereas their outcome strongly depends on the reliability of the input data used as training sets.^19^ Thus, there is significant interest in expanding the amount of available and, above all, reliable data.

In this work, we perform a comprehensive data screening of *K*_OW_ values to evaluate the current reliability of the existing data. Additionally, we apply experimental approaches commonly used in the literature to determine the partition coefficients of four example APIs. Data screening and the comparison of our own results reveal inherent challenges in determining *K*_OW_ values, especially for substances that partially ionize in aqueous media. Based on this analysis, we propose a new data-reduction method to evaluate experimental data. This method extrapolates the measured partition coefficients as a function of pH, rather than as a function of solute concentration. The results of the proposed method are found in very good agreement with the partition coefficients calculated using the thermodynamic model ePC-SAFT^20–23^ that explicitly considers both neutral and charged species.

## 2 Theoretical Background

### 2.1 Definition of the Octanol–Water Partition Coefficient *K*_OW_

The most common property used to characterize the lipophilicity of a substance is the partition coefficient log* P*. It is defined as the logarithm of the ratio of the molar concentrations *c*_*i*_ of a substance in the octanol (org) and the aqueous (aq) phase in liquid–liquid equilibrium (LLE) (see eq 1).^24,25^ Thus, the organic phase consists of octanol saturated with water, and the aqueous phase consists of water saturated with octanol. The partition coefficient log* P* is defined for the neutral species being partitioned between the two phases.

1 $$\log P=\log_{10}\left(\frac{c_{i}^{\mathrm{org}}}{c_{i}^{\mathrm{aq}}}\right)$$

Lipophilic APIs have a positive log *P* value, while hydrophilic APIs have a negative log *P* value.

log *P* is dependent on the overall solute concentration. Its limiting value at solute concentration zero is referred to as the octanol–water partition coefficient *K*_OW_ (see eq 2). Thus, to determine *K*_OW_, partitioning experiments must be performed at different solute concentrations and measured partition coefficients have to be extrapolated to solute concentration zero.^26^

2 $$K_{\mathrm{OW}}={P|}_{c_{i}\to 0}$$

Molar solute concentrations *c*_*i*_ in the organic and aqueous phases can be converted into mole fractions *x*_*i*_ by using the molar volumes *v*_*n*_ of the corresponding phases (eq 3).

3 $$x_{i}=v_{n}\cdot c_{i}$$

Applying the equilibrium conditions for an LLE (eq 4), the ratio of the mole fractions of substance *i* at solute concentration zero equals the inverse ratio of its activity coefficients at infinite dilution γ_*i*_^∞^ in the two phases (eq 5).

4 $$x_{i}^{\mathrm{org}}\cdot \gamma_{i}^{\mathrm{org}}=x_{i}^{\mathrm{aq}}\cdot \gamma_{i}^{\mathrm{aq}}$$

5 $${\frac{x_{i}^{\mathrm{org}}}{x_{i}^{\mathrm{aq}}}|}_{x_{i}\to 0}=\frac{\gamma_{i}^{\infty ,\mathrm{aq}}}{\gamma_{i}^{\infty ,\mathrm{org}}}$$

eqs 1, 2, 3, and 5 result in eq 6, which was used in this work to calculate *K*_OW_ values via thermodynamic modeling. The activity coefficients at infinite dilution γ_*i*_^∞^ and the molar volumes of the two phases were obtained in this work using the thermodynamic model ePC-SAFT.^20–23^

6 $$K_{\mathrm{OW}}=\frac{{v_{n}}^{\mathrm{aq}}}{{v_{n}}^{\mathrm{org}}}\cdot \frac{\gamma_{i}^{\infty ,\mathrm{aq}}}{\gamma_{i}^{\infty ,\mathrm{org}}}$$

### 2.2 Experimental Determination of Partition Coefficients

Figure 1 shows the LLE of a ternary system containing water, an organic solvent, and a solute. For an organic solvent/water ratio of 1:1, different overall solute concentrations lead to demixing along different tie lines, e.g., T1 and T2, resulting in different solute concentrations in the two phases. Thus, the log *P* value depends on the solute concentration and measuring partitioning at just one solute concentration is not sufficient to determine the *K*_OW_ value.

**Figure 1:**
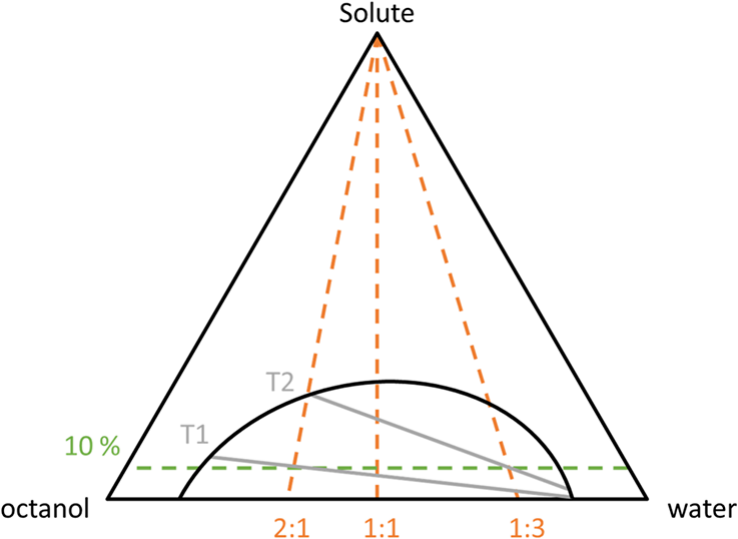
Schematic representation of a ternary LLE of water, octanol, and a solute. The phase boundary is shown as the black line. 10% solute concentration is indicated as the green dashed line. The tie lines are gray solid lines. Different ratios of the organic solvent and water are indicated as orange dashed lines.

To obtain *K*_OW_ values, the solute concentrations in the two phases must be measured at several overall solute concentrations, and partition coefficients then need to be extrapolated to solute concentration zero. For that purpose, it is important to use low solute concentrations for the measurements. The OECD recommends an upper limit of 0.01 mol L^–1^ in each phase^7^ to be as close as possible to the state of solute concentration zero.

According to the OECD guidelines, measurements should also be taken for different volume ratios of the two solvents. For a fixed overall solute concentration of, e.g., 10% (green dashed line) and different volume ratios (orange dashed lines), demixing again occurs along different tie lines, resulting in different solute concentration ratios and consequently in different log *P* values. However, this difference is only significant at solute concentrations way above solute concentration zero. Thus, the examination of different volume ratios is usually not necessary.

Besides directly measuring the solute concentrations in the two liquid phases, the solubilities *c*_*i*_^SLE^ of the solute in pure octanol and in pure water are frequently used as a measure for solute partitioning between octanol and water (see eq 7).

7 $$\log P^{\mathrm{SLE}}=\log_{10}\frac{c_{i}^{\mathrm{SLE},\mathrm{org}}}{c_{i}^{\mathrm{SLE},\mathrm{aq}}}$$

This method requires less experimental effort than partition measurements but provides a value that does not at all correspond to the *K*_OW_ value. By definition, the *K*_OW_ value is the solute concentration ratio (1) in the ternary system solute/octanol/water and (2) at solute concentration zero. Neither one nor the other applies when measuring solute solubilities in the pure liquids. This topic will be discussed again later in this work (see Section 5.2).

### 2.3 Partitioning of Ionizable Substances

The considerations in the following Sections 2.3 and 2.4 refer to acidic substances but can also be applied analogously to basic substances.

When dealing with the *K*_OW_ value of pharmaceuticals, it is important to note that approximately 95% of the APIs are ionizable.^27^ This drastically changes their hydrophilicity, as well as their distribution between organic and aqueous phases. The distribution of ionizable substances is described via the so-called distribution coefficient log *D* (eq 8). The log *D* value not only considers the neutral species but also the charged species in the two phases.^24^ For nonionizable substances, the values log *D* and log *P* are the same.

8 $$\log D=\log_{10}\left(\frac{c_{i,\mathrm{neutral}}^{\mathrm{org}}+c_{i,\mathrm{charged}}^{\mathrm{org}}}{c_{i,\mathrm{neutral}}^{\mathrm{aq}}+c_{i,\mathrm{charged}}^{\mathrm{aq}}}\right)$$

Similar to the log *P* value, the log *D* value depends on the overall solute concentration. At solute concentration zero and for octanol as the organic solvent, eq 9 is obtained.

9 $$D_{\mathrm{OW}}={D|}_{c_{i}\to 0}$$

eq 10 is used to calculate *D*_OW_ using the molar volumes *v*_*n*_ of the two phases as well as the solute activity coefficients at infinite dilution in the two phases (see Supporting Information).

10 $$D_{\mathrm{OW}}=\frac{v_{n}^{\mathrm{aq}}}{v_{n}^{\mathrm{org}}}\cdot \sqrt{\frac{\gamma_{i,\mathrm{charged}}^{\infty ,\mathrm{aq}}}{\gamma_{i,\mathrm{charged}}^{\infty ,\mathrm{org}}}\cdot \frac{\gamma_{{H_{3}O}^{+}}^{\infty ,\mathrm{aq}}}{\gamma_{{H_{3}O}^{+}}^{\infty ,\mathrm{org}}}}$$

Most notably, the *D*_OW_ value does not depend on the properties of the neutral species. This is because, at solute concentration zero, a substance completely ionizes.^28^ Therefore, the neutral species is no longer present and thus does not influence the *D*_OW_ value. Calculated *D*_OW_ values obtained this way therefore only depend on how accurately a thermodynamic model describes the activity coefficients of the charged species and of the corresponding counterion at solute concentration zero. Since ionization is significantly reduced in organic solvents,^29^ it is reasonable to assume that the organic phase contains only a small amount of the charged species. Thus, the log* P* value is the upper limit of the log *D* value (compare eqs 1 and 8 as well as Figure 3).

Figure 2b shows example data of log* D* values as a function of overall solute concentration *c*_*i*_. As can be seen, the experimental data approach the *y*-axis asymptotically (see also Section 2.4). When only considering the data at low solute concentrations, as suggested by the OECD guidelines^7^ and extrapolating to solute concentration zero log* D*_OW_ = 1.81 (Figure 2a) is obtained. Extrapolating the data of the same system obtained at higher solute concentrations results in log *D*_OW_ = 2.41 (Figure 2c).

**Figure 2:**
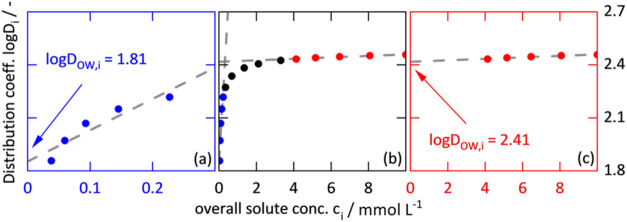
Schematic diagrams of the distribution coefficient log *D* (dots) as a function of the overall solute concentration *c*_*i*_. (a–c) show the same data, but focus on different solute concentration ranges. The gray dashed lines represent linear extrapolations to a solute concentration of zero.

Obviously, the overall solute concentration used for the measurements and its extrapolation are decisive for the log *D*_OW_ value obtained.

### 2.4 Influence of pH and *pK*_a_ on Solute Partitioning

An acidic substance AH dissociates in water according to eq 11 into its charged form *A* ^–^ and a hydronium ion H_3_O^+^. This reaction is described by the thermodynamic acid constant *pK*_a_.

11 $$\mathrm{AH}+H_{2}O\overset{pK_{a}}{↔}A^{-}+{H_{3}O}^{+}$$

The *pK*_a_ value (eq 12) is usually defined by the molar concentrations *c*_*i*_ of the species participating in the dissociation reaction (excluding water).

12 $$pK_{a}=-\log_{10}\left(\frac{c_{A^{-}}\cdot c_{H_{3}O^{+}}}{c_{\mathrm{AH}}}\right)$$

*c*_H_3_O_^+^ is directly connected to pH according to eq 13.^30^

13 $$\mathrm{pH}=-\log_{10}\left(c_{H_{3}O^{+}}\right)$$

Using the dissociation constant *pK*_a_ and pH, experimentally determined *D* values can be converted into *P* values applying eq 14.^31,32^ It is important to note that eq 14 is a simplification that holds for small species concentrations. The derivation and the applied assumptions can be found in the Supporting Information.

14 $$P=D\cdot \left(1+10^{\mathrm{pH}-{\mathrm{pK}}_{a}}\right)$$

Figure 3 schematically shows the development of log *D* values and log *P* values for an acidic substance as a function of its overall concentration *c*_*i*_ as obtained from eqs 1 and 8. As mentioned above, log *D* values exhibit strong nonlinear behavior at low solute concentrations, whereas the log *D*_OW_ value even diverges, while log *P* values follow an almost linear trend. As can be seen, log *P* is the upper limit for log *D* (see Section 2.3).

**Figure 3:**
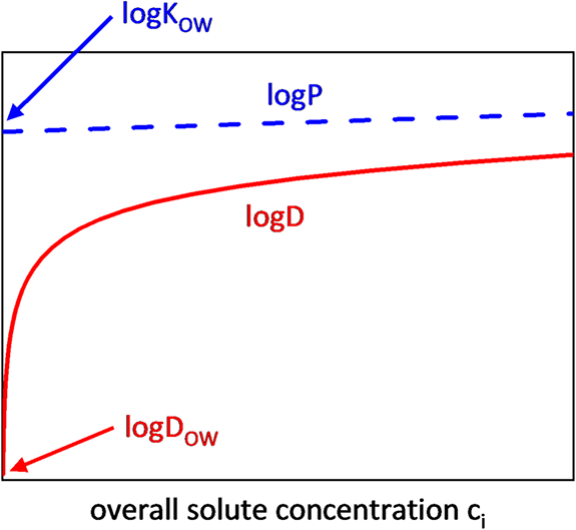
Schematic diagram of the distribution coefficient log *D* (red line) and the partition coefficient log *P* (blue dashed line) against the overall solute concentration *c*_*i*_. log *D*_OW_ and log *K*_OW_ can be found at solute concentration zero.

For ionizable solutes, only the log *D* value is experimentally accessible, since the usually applied analytical techniques cannot distinguish between charged and neutral species.^33^ Thus, the log *P* value can only be determined by modeling (using eq 1) or measured after adding excipients that change pH and thus change the degree of ionization of the target substance to a minimum. However, adding excipients contradicts the definition of log *P*, which is defined as the partition coefficient in the ternary mixture containing the target substance, octanol, and water (see Section 2.1).^34–40^

## 3 Literature Data Screening for *K*_OW_ Values of APIs

Table 1 lists the literature values of partition coefficients or distribution coefficients for various APIs. All values listed in Table 1 were obtained from LLE measurements in the API/octanol/water system. The column “property stated” indicates the type of property that was specified by the corresponding authors as measured in their works. The column “property reported” indicates the property that was actually measured according to the description of the experiments in the papers. The third column indicates how the reported values were determined: experimentally (exp.), converted from the experimental log *D* values, i.e., using eq 14 or similar (conv.) or calculated using a theoretical tool (calc.). If the pH of the aqueous phase was known, it is given in brackets. If the referenced works did not clearly state how the reported values were determined, this is indicated as “N/A”. If known, solute-concentration ranges of the measurements and the ionic strength are given in the fifth and sixth columns, respectively. Otherwise, it is indicated as “N/A”. The literature values, which we consider being the most reliable ones given the conditions of their estimation are marked in bold (see also Section 5.2).

**Table 1:** log *P*, log *D*, log *K*_OW_, and log *D*_OW_ Values of Different APIs Reported in the Literature as well as the Determination Method Used (Experimental, Converted, or Calculated)^a^

API	property stated	property reported	method	value	concentration / mmol L^–1^	ionic strength / mmol L^–1^	ref
carbamazepine^b^	**log *P***	**log *P***	**exp. (pH 7)**	**1.40**	**N/A**	**0.1**	^34^
	**log *P***	**log *P***	**exp.**	**1.90**	**N/A**	**N/A**	^34^
	log *K*_OW_	log *K*_OW_	calc.	2.45	0	0	^35^
felodipine^b^	**log *P***	**log *P***	**exp.**	**3.86**	**N/A**	**N/A**	^36^
	log *P*	N/A	N/A	5.58	N/A	N/A	^37^
fenofibrate^b^	log *P*	N/A	N/A	4.60	N/A	N/A	^38^
	log *P*	N/A	N/A	5.20	N/A	N/A	^39^
	log *P*	N/A	N/A	5.80	N/A	N/A	^40^
griseofulvin^b^	**log *P***	**log *P***	**exp. (pH 7.4)**	**1.98**	**1.6**	**0.15**	^41^
	log *P*	N/A	N/A	2.15	N/A	N/A	^42^
	log *P*	N/A	N/A	2.36	N/A	N/A	^43^
cinnarizine	log *P*	N/A	N/A	5.60	N/A	N/A	^44^
	log *P*	N/A	N/A	5.71	N/A	N/A	^37^
	log *P*	N/A	N/A	5.80	N/A	N/A	^45^
itraconazole	log *K*_OW_	N/A	N/A	5.66	N/A	N/A	^46^
	log *P*	N/A	N/A	6.20	N/A	N/A	^47^
lidocaine	log *D*_OW_	log *D*	exp. (pH 7.4)	1.63	N/A	0.15	^48^
	**log *P***	**log *P***	**exp. & conv.**	**2.45**	**N/A**	**0.15**	^11^
	log *P*	N/A	N/A (pH 11.2)	3.40	N/A	0	^49^
ritonavir	log *P*	N/A	N/A	0.45	N/A	N/A	^50^
	log *P*	log *D*_OW_	calc.	1.54	0	0	^51^
	log *P*	N/A	N/A (pH 6.8)	4.30	N/A	0.1	^52^
terfenadine	log *P*	log *D*	exp.	4.47	N/A	0.15	^34^
	**log *P***	**log *P***	**exp (pH 12)**	**4.96**	**N/A**	**0.1**	^34^
	log *P*	log *D*	exp.	6.08	N/A	N/A	^34^
thiabendazole	log *K*_OW_	log *D*	exp.	1.94	1	0.75	^53^
	log *K*_OW_	log *K*_OW_	calc.	2.30	0	0	^53^
	log *K*_OW_	N/A	N/A (pH 6)	2.47	N/A	N/A	^53^
	**log *P***	**log *D***	**exp.**	**2.55**	**0.01**	**0**	^54^
artemisinin	log *P*	log *D*_OW_	calc.	1.72	0	N/A	^55^
	log *P*	N/A	N/A	2.94	N/A	N/A	^56^
celecoxib	log *P*	N/A	N/A	3.50	N/A	0.16	^57^
	log *P*	log *D*_OW_	calc.	3.68	0	0	^58^
	log *P*	log *P*	exp. (pH 2)	3.90	N/A	0.1	^34^
	**log *D***	**log *D***	**exp. (pH 7.4)**	**4.30**	**0.02**	**0.1**	^59^
glibenclamide	log *P*	N/A	N/A	0.30	N/A	N/A	^60^
	log *P*	N/A	N/A	3.08	N/A	N/A	^37^
	log *P*	N/A	N/A	4.80	N/A	N/A	^61^
ibuprofen	**log *D*_OW_**	**log *D***	**exp. (pH 7.4)**	**1.00**	**6.4**	**0**	^62^
	**log *K*_OW_**	**log *P***	**exp. & conv.**	**2.48**	**2.4**	**0**	^63^
	log *P*	log *P*	exp. & conv.	3.97	N/A	0.15	^11^
indomethacin	log *P*	N/A	N/A	3.51	N/A	N/A	^37^
	log *P*	log *P*	exp. (pH 2)	3.89	N/A	0.15	^34^
	log *P*	N/A	N/A	4.27	N/A	N/A	^64^
naproxen	**log *D***	**log *D***	**N/A (pH 7.4)**	**0.33**	^c^	**0.15**	^65^
	log *P*	N/A	N/A (pH 5)	2.38	N/A	0	^66^
	log *K*_OW_	N/A	N/A	3.18	N/A	N/A	^67^
	**log *P***	**log *P***	**N/A (pH 2)**	**3.34**	^c^	**N/A**	^65^
nifedipine	**log *P***	**log *D***	**exp.**	**0.30**	**3.4–12.8^c^**	**0**	^68^
	log *K*_OW_	log *D*	exp. (pH 7.4)	2.36	N/A	0	^69^
	log *K*_OW_	log *D*_OW_	calc.	3.17	0	0	^35^
paracetamol	log *K*_OW_	N/A	N/A	0.48	N/A	N/A	^70^
	**log *D*_OW_**	**log *D***	**exp. (pH 7.4)**	**0.78**	**12.7**	**0**	^62^
	log *K*_OW_	N/A	N/A	3.02	5	N/A	^71^

a A pH shift is specified in brackets. If the solute concentration is known, it is specified in the fifth column. If the ionic strength is known, it is specified in the sixth column. All values in this table were determined based on the LLE method and were determined at temperatures between 20 °C and 30 °C. We consider the values marked in bold as most reliable given the conditions of their estimation.

b These APIs are nonionizable.

c The reported value is the average of all measurements in the indicated solute concentration range, if given.

First, it is notable that a large portion of information in Table 1 was not available from the referenced publications. Moreover, for most properties (∼80%), we observed a discrepancy between the properties stated and the property reported. In some cases, it was not even possible to assign which property was measured. “N/A” in the fifth column indicates that no solute concentration was specified in the referenced work. When specified, the solute concentrations used for the measurements were, in most cases, very low. The latter is very positive because this ensures a certain proximity to solute concentration zero, which is important for values referred to as log *K*_OW_ or log *D*_OW_ (see Section 2.3).

For nonionizable APIs (top four APIs of Table 1), deviations between reported log* P* values for the same substance are expected to be smaller than for the ionizable APIs, as the main error source (API ionization, see Section 2.3) does not exist. However, even for these nonionizable APIs, the highest published log *P* value is on average 1.3 units higher than the lowest. The main reason for this certainly is the very low concentration of the very hydrophobic APIs in the aqueous phase, which can be measured only with high experimental uncertainties. An analogous screening for approximately 500 liquid solutes (see Supporting Information for examples)^31^ revealed an average deviation between the lowest and highest published value of about 0.8 units. This appears to be the best accuracy currently achievable.

Considering the deviations found for ionizable APIs, we first note the much greater scatter of the data. The largest deviation between two reported literature values was found for glibenclamide with a difference of 4.5 units, a deviation by 4 orders of magnitude for the same substance. On average, the lowest and highest published values of an ionizable API differ by 3.5 units, which is still a deviation by 3 orders of magnitude. This corresponds to a deviation more than hundred times larger than that found for nonionizable APIs.

Ionic strength affects both the partitioning coefficient and distribution coefficient, which typically increase with increasing salt concentration (usually termed salting out). However, an ionic strength of 0.15 M results in an increase of log *D* values by at most 0.2 logarithmic units compared with measurements without salt.^72,73^ Therefore, the deviations observed in Table 1 cannot be exclusively attributed to the different ionic strengths used in the measurements.

## 4 Experimental Section

### 4.1 Materials

In this work, we investigated the four example APIs: naproxen (acidic), ibuprofen (acidic), lidocaine (basic), and griseofulvin (nonionizable). All APIs were used as received without further purification. Ultrapurified water (Merck Millipore, Darmstadt, Germany) and 1-octanol were used for the log *D* measurements. All relevant data of the chemicals used are given in Table 2.

**Table 2:** APIs and Other Chemicals Used in This Work, Their CAS, Purity, and Manufacturer as well as Their Extinction Maximum and *pK*_a_ ^a^

substance	CAS	purity/wt %	manufacturer	extinction maximum/nm	*pK*_a_/-
water	7732–18–5	100	Merck Millipore		
octanol	111–87–5	>99	Sigma-Aldrich		
naproxen	22204–53–1	>99	TCI	271	4.18 (25 °C; 0.15 M)^74^
ibuprofen	15687–27–1	>98	TCI	223	4.42 (25 °C; 0.15 M)^74^
lidocaine	137–58–6	>98	Sigma-Aldrich	262	7.92 (22.5 °C; 0.1 M)^75^
griseofulvin	126–07–8	>97	Alfa Aesar	292	

a The temperature and ionic strength used to determine the *pK*_a_ values are given in brackets.

### 4.2 Measuring API Solubilities in Octanol

An excess amount of API was added to octanol and equilibrated for at least 3 days while being shaken at 25 °C in a Thermomixer (Eppendorf, Hamburg, Germany). The API concentrations in the saturated octanol solution were determined by using a vibrating tube densimeter (DMA 4100 M, Anton Paar, Graz).

### 4.3 Determining API Distribution Coefficients

API was added to octanol/water mixtures (volume ratio 1:3) at four different overall API concentrations. The samples were equilibrated for at least 2 days while shaken and tempered at 25 °C in a Thermomixer. After that, the two liquid phases were analyzed via ultraviolet–visible (UV/vis) (SPECORD 210 PLUS, Analytik Jena) to determine the API concentration. pH of the aqueous phase was measured using a pH electrode (GHM Group Greisinger, Remscheid). The experiments were performed in triplicate, and average values are reported in the Supporting Information. The mean average deviation of three measurements was approximately 5%.

### 4.4 Thermodynamic Model ePC-SAFT

In this work, ePC-SAFT^20–23^ was used to calculate the activity coefficients necessary for determining the log *D*_OW_ and log *K*_OW_ values of APIs according to eqs 6 and 10. ePC-SAFT calculates the residual Helmholtz energy *a* ^res^ as a sum of different contributions (eq 15). These contributions account for repulsion *a* ^hc^, attractions via van der Waals forces *a* ^disp^ and associative interactions *a* ^assoc^ via hydrogen bonds. *a* ^DH^ accounts for electrostatic interactions between two ions and *a* ^born^ considers interactions between ions and their surrounding medium. The model parameters used in this work can be found in the Supporting Information.

15 $$a^{\mathrm{res}}=a^{\mathrm{hc}}+a^{\mathrm{disp}}+a^{\mathrm{assoc}}+a^{\mathrm{DH}}+a^{\mathrm{born}}$$

## 5 Results and Discussion

### 5.1 Determination of log *D* and log *P* Values

The distribution coefficients log *D* were determined according to the method described in Section 4.2. The experimental data are shown in Figure 4 as a function of the overall API concentration. The OECD recommended upper limit for the API concentration in each of the two phases (0.01 mol L^–1^) is shown as a dashed line. It is noticeable that there is a characteristic sharp drop of log *D* values as the API concentration decreases. This drop does not exist for the nonionizable griseofulvin.

**Figure 4:**
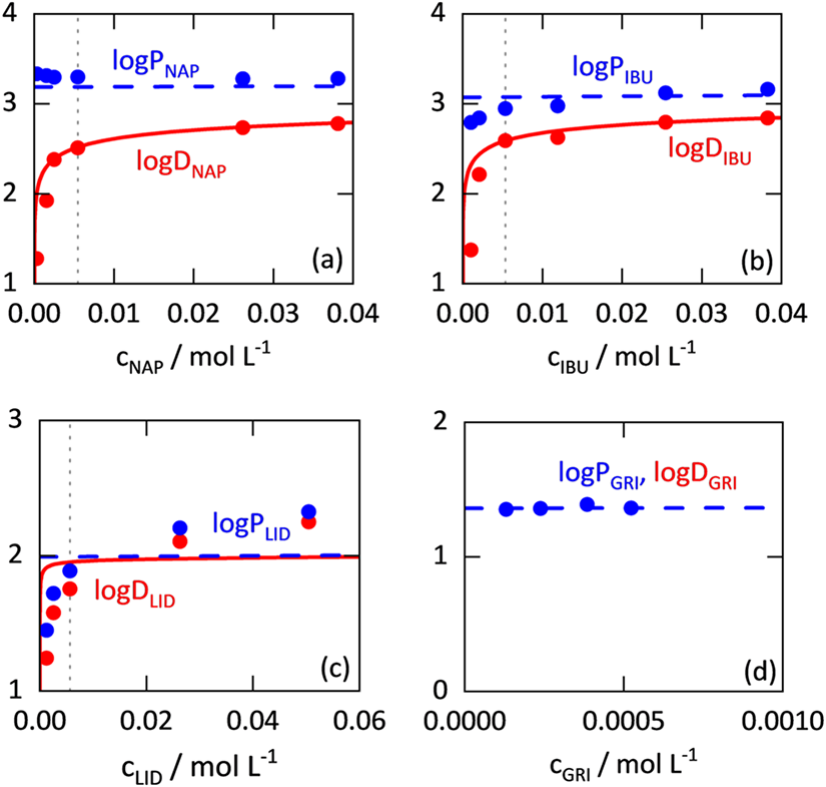
Partition coefficients log *P* (blue, dashed) and distribution coefficients log *D* (red) of APIs (experimental data indicated as circles. Solid lines were predicted with ePC-SAFT). The gray dashed lines mark the OECD limit of 0.01 mol L^–1^ of API in each phase.^8^ Experimental data and standard deviations can be found in the Supporting Information. NAP: naproxen, IBU: ibuprofen, LID: lidocaine, and GRI: griseofulvin.

Figure 4 also shows the log* P* values obtained from the experimentally determined log *D* values by using eq 14. In contrast to log *D* values, log *P* values (also of acidic and basic APIs) depend almost linearly on the API concentration. Due to the higher lidocaine concentration in the aqueous phase (which leads to a lower log *D* value; Figure 4c), the degree of ionization in that phase is significantly lower for lidocaine than for naproxen and ibuprofen (compare Section 2.4). Therefore, the difference between the log *D* and log *P* values of lidocaine is smaller than those of naproxen and ibuprofen. For the nonionizable griseofulvin, the experimentally determined log *D* values are equivalent to the log *P* values (see Figure 4d and Section 2.3).

Figure 4 also shows the log *D* and log *P* values predicted with ePC-SAFT. As can be seen, ePC-SAFT is able to predict these values with an almost quantitative agreement with the experimental data across the entire investigated API concentration range.

### 5.2 Proposed Approaches to Determine log *D*_OW_ and log *K*_OW_ Values

To determine log *D*_OW_ and log *K*_OW_, the log *D* and log *P* values must be extrapolated to a solute concentration of zero. As Figure 4 shows, it is straightforward to extrapolate log *P* values as a function of the overall solute concentration, which leads to the log *K*_OW_ value. However, log *P* values for acidic or basic solutes can be obtained only from experimental log *D* values after conversion using the pH value (eq 14).

log* D* values for those compounds strongly decrease as a function of the overall solute concentration. Thus, extrapolating these values to solute concentration zero is obviously an error-prone method to determine the log *D*_OW_ values. Instead, we propose to extrapolate experimental log *D* values as a function of the pH value of the aqueous phase (Figure 5). This value is easy to measure and required, anyway, to convert log *D* values into log *P* values using eq 14. According to eq 11, the number of hydronium ions corresponds to the number of charged solute species (neglecting the autoprotolysis of water). Plotting log *D* values versus pH of the aqueous phase thus corresponds to plotting these values versus the logarithmic concentration of the charged solute species in the aqueous phase. At very low solute concentrations, the latter in fact corresponds to the overall solute concentration in the aqueous phase, as the solute is almost fully ionized at concentration zero.

**Figure 5:**
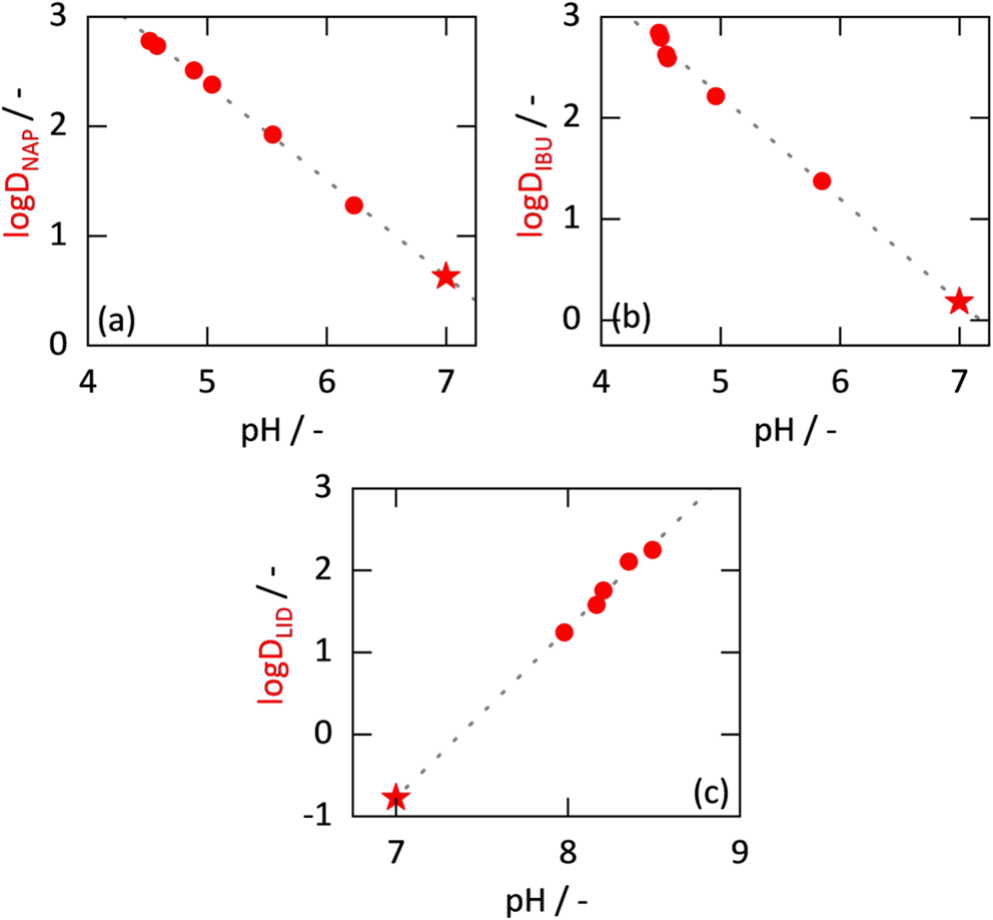
Distribution coefficients log *D* of APIs versus pH of the aqueous phase. The red circles are experimental data. The gray dotted line is a linear trend line. The red star is the log *D*_OW_ value from extrapolation to a pH of 7. NAP: naproxen, IBU: ibuprofen, LID: lidocaine, GRI: griseofulvin.

The decisive advantage of this approach is that pH in the aqueous phase at solute concentration zero has a clearly defined value: it is 7 (the presence of a very small amount of octanol was determined not to cause a noticeable change in pH). Figure 5 shows the measured log *D* values for the three ionizable APIs investigated in this work plotted versus the pH of the aqueous phase (data can be found in the Supporting Information). This leads to a linear trend (see also eq 13) and to a well-defined log *D* value at pH 7, namely the log* D*_OW_ value, indicated by a red star.

The measured pH values of acidic APIs are smaller than 7, while those of basic APIs are larger than 7. As the API concentration decreases, pH approaches 7 in both cases. Using the proposed approach to evaluate the experimental data, the log *D*_OW_ value can be read off as the ordinate intercept at pH 7. It should be noted that this approach naturally does not apply for nonionizable solutes, as they practically do not change pH. However, the log *K*_OW_ value of these solutes can be easily determined by extrapolating the log *P* values to the solute concentration zero as described above (see also Figure 4).

We thus suggest two methods for experimentally determining log *K*_OW_ values (Figure 6). Method 1: plot the log *D* values against pH of the aqueous phase and extrapolate to pH value of 7 to obtain the log *D*_OW_ value. This can then be converted to the log *K*_OW_ value using eqs 2 and 14. Method 2: use pH to convert the measured log *D* values into log *P* values using eq 14. Then log *P* values are extrapolated to solute concentration zero to obtain the log *K*_OW_ value.

**Figure 6:**
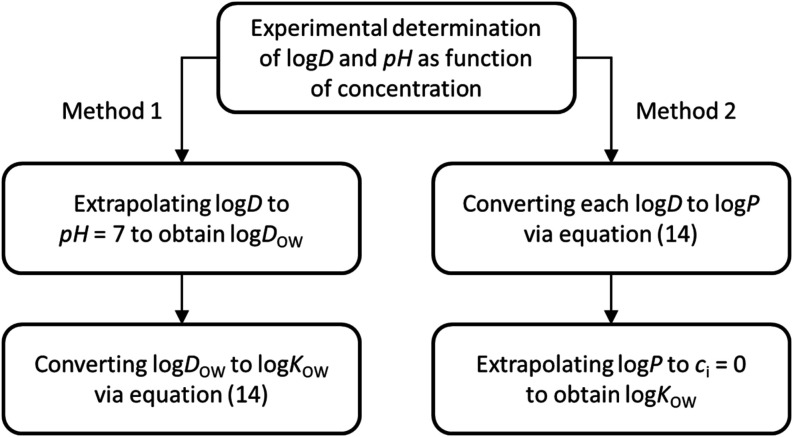
Two methods are proposed to correctly determine log *K*_OW_ values experimentally.

In addition, log *K*_OW_ values can also be determined by applying a thermodynamic model using eq 6. This approach does not require any extrapolations. Instead, log *K*_OW_ values are directly calculated from the activity coefficients at infinite dilution in the corresponding liquid equilibrium phases (see eq 6).

Table 3 summarizes the log *K*_OW_ values for the four APIs determined in this work using Method 1, Method 2, and Method 3. It also contains the log *P* ^SLE^ values obtained from the ratios of the API solubilities in pure octanol and pure water (eq 7 and Section 4.1; results can be found in the Supporting Information). For comparison, the most reliable values from Table 1 are also included in Table 3.

**Table 3:** Comparison of log *K*_OW_ Values at 25 °C Obtained Using Different Methods Investigated in This Work and the log *P* ^SLE^ Value Calculated as the Ratio of the Solubilities in Pure Octanol and in Pure Water^a^

API	log *P* ^SLE^/-	method 1	method 2	method 3	literature value
naproxen	2.57	3.45	3.31	3.08	3.34 ^65^
ibuprofen	4.42	2.73	2.84	2.95	2.48 ^63^
lidocaine	2.09	0.30	1.70	1.96	2.45 ^11^
griseofulvin	2.26		1.34	1.34	1.98 ^41^

a Moreover, the most reliable literature values from Table 1 are included.

First, the ratio of the API solubilities in pure octanol and in pure water (log *P* ^SLE^ value) does not even approximately match the log *K*_OW_ values. This confirms the statement above that the ratio of the solute solubilities in octanol and water does not reasonably approximate the partition coefficient in the ternary system (see Section 2.2). In contrast, the three methods proposed and used in this work provide very similar log *K*_OW_ values.

A comparison of Method 1 and Method 2 (Figure 6) reveals that the two values obtained for naproxen and ibuprofen are very close. The difference between the highest and lowest values is only 0.14 units for naproxen and 0.11 units for ibuprofen. These uncertainties are much smaller than the average difference of 1.3 units for nonionizable APIs from Table 1 and even smaller than the average difference of 0.8 units found for other neutral solutes (see Supporting Information). The difference for lidocaine is 1.4 units, which is the same as that for neutral solutes. The values from Method 1 and Method 2 are also in the same order of magnitude as the most reliable literature values in Table 1. When comparing the difference between the highest and the lowest values for the ionizable APIs naproxen, ibuprofen, and lidocaine with the same values for the ionizable APIs in Table 1 (on average 3.5 units), we see that there is still a hundredfold improvement.

The log *K*_OW_ values predicted by ePC-SAFT (Method 3) show very good agreement with the experimentally determined ones. For griseofulvin, this value was even quantitatively predicted. This is particularly remarkable as model parameters for ePC-SAFT were not fitted to the log *K*_OW_ values, and thus, the latter were fully predicted.

The accuracy and reproducibility of determining log *K*_OW_ values for ionizable APIs could thus be remarkably improved using any of the three methods proposed in this work, each of which fully compensates for the error source caused by extrapolation of partition coefficients to solute concentration zero.

## 6 Conclusions

In this work, we proposed two different data-reduction methods and one theoretical method to resolve the uncertainty in determining octanol–water partition coefficients.

A literature survey of API partition coefficients revealed a large scatter of data showing deviations of up to 4 orders of magnitude for the same substance. The greatest deviation was found for acidic or basic APIs that ionize in water (difference of 3.5 logarithmic units between the highest and the lowest values vs 1.3 units for nonionizable APIs). This work identified extrapolating the experimentally determined distribution coefficients to solute concentration zero as the main reason for the large scatter in *K*_OW_ values of acidic or basic compounds.

To solve this problem, we propose extrapolating experimentally determined distribution coefficients as a function of easy-to-determine pH instead of the overall solute concentration. This approach of evaluating experimental data offers a straightforward but highly reliable possibility of determining *K*_OW_ values. A second meaningful option is to convert experimental distribution coefficients into partition coefficients, which then can easily be extrapolated to solute concentration zero. This conversion also requires the pH value, which should therefore always be measured simultaneously when distribution coefficients.

The two proposed evaluation methods for experimental data provide the same level of accuracy when determining *K*_OW_ values for both ionizable APIs and nonionizable APIs (difference of 0.5 logarithmic units between the highest and the lowest values in this work vs 3.5 logarithmic units in literature) and completely eliminate the error source of ionization.

Finally, we theoretically predicted *K*_OW_ and *D*_OW_ values from solute activity coefficients at infinite dilution using the thermodynamic model ePC-SAFT. On average, the difference between the predicted and experimentally determined *K*_OW_ values was only 0.36 logarithmic units. For griseofulvin, we were able to predict the *K*_OW_ value.

The proposed data-evaluation methods can easily be implemented without great additional effort and used together with any existing analytical method, ensuring a standardized and reliable database for octanol–water partition coefficients.

## Supplementary Material



## Notes

First, the binary miscibility gap between octanol and water from various experimental literature sources are compared with the modeled miscibility gap using ePC-SAFT; additionally, log *P* values for octanol and water are provided; then, detailed derivations for the theoretical calculation of the distribution coefficient *D*_OW_ at solute concentration zero as well as a simplified conversion of distribution coefficients into partition coefficients are given; next, a list of 19 example liquid organic solvents and their published range of log *P* values is provided to demonstrate that the crux of determining partition coefficients extends beyond the field of pharmaceutical sciences; afterward, we present experimental log *D* and pH data along with measured API solubilities in octanol; additionally, we provide the parameters necessary for modeling the systems considered in this work with ePC-SAFT (PDF)
