# Supplementary material for: The Hidden Crux of Correctly Determining Octanol–Water Partition Coefficients
Source: Mol Pharm. 2025 Jul 3;22(8):4930–9. doi: 10.1021/acs.molpharmaceut.5c00552 (PMC12326361; doi:10.1021/acs.molpharmaceut.5c00552)
Supplement: Supplementary file 1 [file mp5c00552_si_001.pdf]

The hidden crux of correctly determining octanol-water partition coefficients

Supporting Information

Espen Fritschka[1,2], *Gabriele Sadowski*[1,2*]

[1]TU Dortmund University, Department of Biochemical and Chemical Engineering,

Laboratory of Thermodynamics, Emil-Figge-Straße 70, D-44227 Dortmund, Germany

[2]Research Center Chemical Sciences and Sustainability,

Research Alliance Ruhr, D-44780 Bochum, Germany

Mutual solubilities of octanol and water

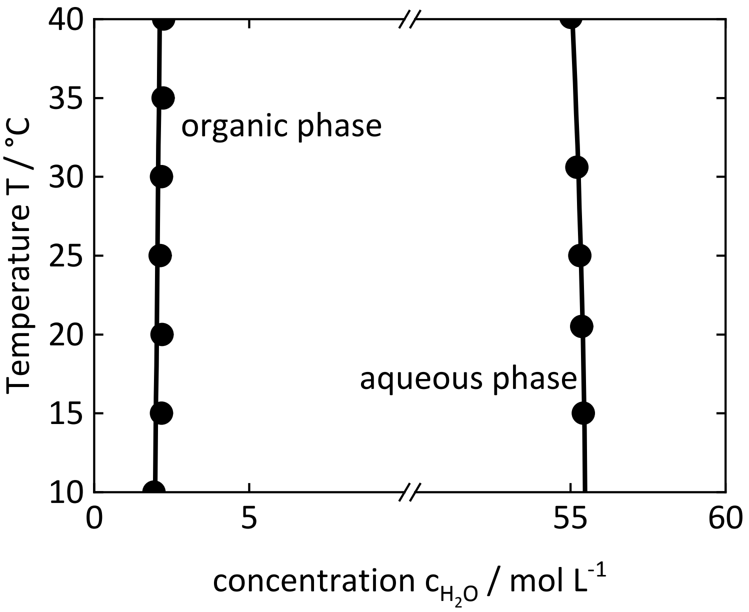

Figure 1: Binary LLE between water and 1-octanol at different temperatures. The lines are modeled with ePC-SAFT. The experimental data (circles) were taken from various literature sources [1–4].

The mutual solubilities of octanol and water is described with excellent agreement to the experimental data. It can also be used to calculate the partition coefficients log*P* for water and octanol in the octanol/water binary system as given in Table 1. The average deviation in the log*P* values between the different literature sources is less than 3%. The mutual solubilities of octanol and water lead to a water-rich phase containing about 0.005 wt% octanol and an octanol-rich phase containing about 4.5 wt% water at 25°C.

Table 1: Averaged mutual solubilities c_i of water and octanol in the aqueous and organic phase between 10 °C and 40 °C and the resulting log*P*.

Substance	c_i^{aq} / mol L^{-1}	c_i^{org} / mol L^{-1}	$\log P$ / —
water	55.2721	2.1301	-1.40
octanol	0.0042	6.0542	3.16

Calculation of the octanol-water distribution coefficient D_{OW}

The following derivations are shown for acidic compounds but can also be done for basic substances analogous with OH^- instead of H_3O^+ as the counter-ion.

Equation (1) describes the distribution coefficient at solute concentration zero.

$$D_{OW} = \left. \frac{c_{i,neutral}^{org} + c_{i,charged}^{org}}{c_{i,neutral}^{aq} + c_{i,charged}^{aq}} \right|_{c_{API} \to 0} \tag{1}$$

At solute concentration zero , the degree of ionization α_i approaches 1, i.e. almost only charged species at very low solute concentrations are present . Therefore, only the charged species need to be considered.

$$\alpha_i = \frac{x_{i,charged}}{x_{i,neutral} + x_{i,charged}} \xrightarrow{x_i \to 0} 1 \tag{2}$$

$$D_{OW} \xrightarrow{x_i \to 0} \left. \frac{c_{i,charged}^{org}}{c_{i,charged}^{aq}} \right|_{c_{API} \to 0} \tag{3}$$

Using the isofugacity criterion for a charged solute species results in equation (4). The corresponding counter-ion has to be considered as well.

$$x_{i,charged}^{org} \cdot \gamma_{i,charged}^{org} \cdot x_{H_3O^+}^{org} \cdot \gamma_{H_3O^+}^{org} = x_{i,charged}^{aq} \cdot \gamma_{i,charged}^{aq} \cdot x_{H_3O^+}^{aq} \cdot \gamma_{H_3O^+}^{aq} \tag{4}$$

Neglecting the autoprotolysis of water, the hydronium ion concentration $x_{H_3O^+}$ corresponds to the concentration of the charged solute species $x_{i,charged}$.

$$x_{i,charged} = x_{H_3O^+} \tag{5}$$

Substituting equation (5) into equation (4) results after rearrangement in equation (6).

$$\left. \frac{x_{i,charged}^{org}}{x_{i,charged}^{aq}} \right|_{x_{API} \to 0} = \sqrt{\frac{\gamma_{i,charged}^{\infty,aq}}{\gamma_{i,charged}^{\infty,org}} \cdot \frac{\gamma_{H_3O^+}^{\infty,aq}}{\gamma_{H_3O^+}^{\infty,org}}} \tag{6}$$

If we now replace the mole fractions with molar concentrations and use equation (3), we obtain the final equation (7) for the distribution coefficient D_{OW}.

$$D_{OW} = \frac{v_n^{aq}}{v_n^{org}} \cdot \sqrt{\frac{\gamma_{i,charged}^{\infty,aq}}{\gamma_{i,charged}^{\infty,org}} \cdot \frac{\gamma_{H_3O^+}^{\infty,aq}}{\gamma_{H_3O^+}^{\infty,org}}} \tag{7}$$

Derivation of the simplified conversion of partition and distribution coefficient

The concentration-based dissociation constant K_a (which also contains the concentration of water) is defined via equation (8).

$$K_a = \frac{c_{A^-} \cdot c_{H_3O^+}}{c_{AH}} \tag{8}$$

Using the degree of ionization $\left(\alpha_{AH} = \frac{c_{A^-}}{c_{AH}+c_{A^-}}\right)$, the overall concentration of the acid $(c_{0,AH} = c_{AH} + c_{A^-})$, and neglecting the autoprotolysis of water $(c_{H_3O^+} = c_{A^-})$, one obtains equation (9).

$$K_a = \frac{\alpha_{AH}^2}{1-\alpha_{AH}} \cdot c_{0,AH} \tag{9}$$

This is used to calculate the degree of ionization:

$$\alpha_{AH} = \frac{-K_a + \sqrt{K_a^2 + 4 \cdot K_a \cdot c_{0,AH}}}{2 \cdot c_{0,AH}} \tag{10}$$

pH is calculated from this using the HENDERSON-HASSELBALCH equation (11).

$$pH = pK_a + \log_{10}\left(\frac{c_{A^-}}{c_{AH}}\right) = pK_a + \log_{10}\left(\frac{\alpha_{AH}}{1-\alpha_{AH}}\right) \tag{11}$$

From the definitions of *P* and *D*, the following equations (12) and (13) are obtained using the assumption that the organic phase does not contain charged species $\left(c_{A^-}^{org} = 0\right)$.

$$c_{AH}^{org} = P \cdot c_{AH}^{aq} \tag{12}$$

$$D = \frac{c_{AH}^{org}}{c_{AH}^{aq} + c_{A^-}^{aq}} \tag{13}$$

The concentration of the charged species in the aqueous phase $c_{A^-}^{aq}$ is calculated using the dissociation constant (see equation (14)).

$$c_{A^-}^{aq} = K_a \cdot \frac{c_{AH}^{aq}}{c_{H_3O^+}^{aq}} \tag{14}$$

Using equations (12) and (14) and the definitions of *pH* and pK_a in equation (13) gives equation (15), which can be used to convert distribution coefficients into partition coefficients.

$$P = \frac{D \cdot \left(c_{AH}^{aq} + K_a \cdot \frac{c_{AH}^{aq}}{c_{H_3O^+}^{aq}} \right)}{c_{AH}^{aq.}} = D \cdot (1 + 10^{pH - pK_a}) \tag{15}$$

Examples for partition coefficients of liquid solutes

Table 2: Lowest, highest and recommended log*P* values for example compounds [5].

Component	Lower limit	Upper limit	Recommended value
Hexane	3.00	4.20	3.00
Heptane	3.27	4.76	4.50
Methanol	-0.82	-0.32	-0.74
Ethanol	-.032	-0.15	-0.30
Butanol	0.32	1.02	0.84
Acetone	-0.48	-0.24	-0.24
Methyl ethyl ketone	0.26	0.69	0.29
Benzaldehyde	1.43	2.33	1.48
Acetic acid	-0.23	-0.17	-0.17
Diethyl ether	0.77	0.89	0.89
Benzene	1.56	2.34	2.13
Toluene	2.11	2.94	2.73
Phenol	0.70	2.20	1.51
Acetophenone	1.58	1.80	1.63
Chlorobenzene	1.23	2.03	1.87
Aniline	2.18	3.08	2.84
Nitrobenzene	0.85	1.08	0.90
Amphetamine	1.70	1.88	1.85
Pyridine	-1.15	2.05	1.76

SANGSTER [5] denoted "recommended values" for a whole variety of substances, which is still used by the OECD [6] today. He selected recommended values individually for each substance by evaluating the methods used and the values obtained. However, he did not propose a general method for determining the recommended values.

Experimental data of the distribution coefficient measurements

Table 3: Naproxen (NAP) concentrations c_{NAP}, pH of aqueous phase, distribution coefficient D_{NAP} and their standard deviations (SD) measured at 25°C in this work (see section 4.2).

c_{NAP} / mM	pH / -	D_{NAP} / -	SD c_{NAP} / mM	SD pH / -	SD D_{NAP} / -
38.1	4.52	601	3.42	0.003	14.47
26.2	4.58	541	2.21	0.003	23.35
5.5	4.89	324	0.45	0.005	10.21
2.5	5.04	239	0.27	0.004	8.32
1.6	5.55	84	0.13	0.004	4.36
0.3	6.23	19	0.03	0.004	2.23

Table 4: Ibuprofen (IBU) concentrations c_{IBU}, pH of aqueous phase, distribution coefficient D_{IBU} and their standard deviations (SD) measured at 25°C in this work (see section 4.2).

c_{IBU} / mM	pH / -	D_{IBU} / -	SD c_{IBU} / mM	SD pH / -	SD D_{IBU} / -
38.3	4.48	693	2.42	0.005	15.88
25.5	4.50	623	1.18	0.004	17.18
11.9	4.55	420	0.81	0.004	18.39
5.4	4.56	388	0.49	0.003	10.85
2.1	4.96	164	0.13	0.005	5.54
1.0	5.85	24	0.07	0.004	1.72

Table 5: Lidocaine (LID) concentrations c_{LID}, pH values of aqueous phase, distribution coefficient D_{LID} and their standard deviations (SD) at 25°C in this work (see section 4.2).

c_{LID} / mM	pH / -	D_{LID} / -	SD c_{LID} / mM	SD pH / -	SD D_{LID} / -
50.5	8.49	178	3.92	0.004	2.30
26.3	8.35	127	2.85	0.005	6.39
5.7	8.21	57	1.01	0.004	2.59
2.6	8.17	38	0.20	0.004	2.31
1.3	7.98	18	0.03	0.003	2.46

Table 6: Griseofulvin (GRI) concentrations c_{GRI}, partition coefficient P_{GRI} and their standard deviations (SD) measured at 25°C in this work (see section 4.2).

c_{GRI} / mM	P_{GRI} / -	SD c_{GRI} / mM	SD P_{GRI} / -
0.5	23	2.42	1.75

0.4	24	1.18	1.82
0.2	23	0.81	1.54
0.1	23	0.49	1.03

ePC-SAFT parameters used in this work

We used mixing rules and combining rules by BERTHELOT-LORENTZ [7] and WOLBACH and SANDLER [8], respectively.

$$\sigma_{ij} = \frac{\sigma_i + \sigma_j}{2} \tag{16}$$

$$u_{ij} = \left(1 - k_{ij}\right) \cdot \sqrt{u_i \cdot u_j} \tag{17}$$

$$\epsilon^{A_iB_j} = \frac{\epsilon^{A_iB_i} + \epsilon^{A_jB_j}}{2} \tag{18}$$

$$\kappa^{A_iB_j} = \sqrt{\kappa^{A_iB_i} \cdot \kappa^{A_jB_j}} \cdot \left(\frac{\sigma_i \cdot \sigma_j}{\sigma_{ij}}\right)^3 \tag{19}$$

The relative permittivity of water [9] and mixing rule for the relative permittivity [10] are given in equations (20) and (21). All substances except water were modeled with a relative permittivity of 8 [11].

$$\epsilon_{r,H_2O} = -105.2 \cdot \ln(T) + 677.48 \tag{20}$$

$$\epsilon_{r,mix} = \sum_{i=1}^{N} x_i \cdot \epsilon_{r,i} \tag{21}$$

Table 7: ePC-SAFT pure-component parameters of water, octanol and the neutral API species used in this work. The charged API species have the same pure-component parameters as the neutral API species. Moreover, their corresponding charge was used for the calculations [12].

Component	m_i^{seg} / −	σ_i / Å	$u_i\, k_B^{-1}$ / K	$\epsilon^{A_iB_i}\, k_B^{-1}$ / K	$\kappa^{A_iB_i}$ / −	N^{assoc} / −	ref.
water	1.2047	*	353.945	2425.700	0.0451	2/2	[13]
octanol	4.3555	3.7145	262.740	2754.800	0.0022	1/1	[14]
naproxen	8.1100	2.9390	229.450	934.200	0.0200	2/2	[15]
ibuprofen	2.5220	4.4320	374.651	879.415	0.0300	2/2	[16]
lidocaine	5.2935	2.5851	155.970	1830.730	0.0200	2/2	[17]
griseofulvin	14.1740	3.3720	221.261	1985.490	0.0200	2/2	[18]

$*\sigma_{H_2O} = 2.7927 + 10.11 \cdot \exp(-0.01775 \cdot T) - 1.417 \cdot \exp(0.01146 \cdot T)$

Table 8: ePC-SAFT pure-component parameters of the ions and binary interaction parameters with water used in this work [19].

Component	m_i^{seg} / −	σ_i / Å	$u_i\, k_B^{-1}$ / K	k_{ion,H_2O} / −
H_3O^+	1	3.4654	500	0.25

Component	m_i^{seg} / −	σ_i / Å	$u_i\, k_B^{-1}$ / K	k_{ion,H_2O} / −
OH^-	1	2.0177	650	-0.25

Table 9: Binary interaction parameters $k_{i,j}$ between the neutral API species and water or octanol used in this work. $k_{i,OcOH}$ were fitted to Table 10 in this work.

Component	k_{i,H_2O}	$k_{i,OcOH}$
naproxen	$2.270 \cdot 10^{-4} \cdot T$ - 0.06120 [20]	0.0230
ibuprofen	$3.527 \cdot 10^{-4} \cdot T$ - 0.01597 [21]	0.0680
lidocaine	0.064 [17]	0.0800
griseofulvin	$2.641 \cdot 10^{-4} \cdot T$ - 0.15500 [18]	0.0666

The temperature dependent binary interaction parameter between water and octanol is $k_{H_2O,OcOH} = 4.91 \cdot 10^{-4} \cdot T - 0.12853$ [22].

Solubilities of APIs in water and in octanol

Table 10: Solubilities of APIs in 1-octanol c_i^{OcOH} and in water $c_i^{H_2O}$ (taken from literature) and at 25 °C. c_i^{OcOH} were determined in this work.

Component	c_i^{OcOH}/ $mol\ L^{-1}$	$c_i^{H_2O}$/ $mol\ L^{-1}$	Ref.
naproxen	0.0045	$2.45 \cdot 10^{-5}$	[23]
ibuprofen	2.1408	$1.74 \cdot 10^{-2}$	[24]
lidocaine	0.1112	$3.00 \cdot 10^{-4}$	[25]
griseofulvin	1.4555	$5.52 \cdot 10^{-5}$	[26]

1. References

(1) Kinoshita, K.; Ishikawa, H.; Shinoda, K. Solubility of Alcohols in Water Determined the Surface Tension Measurements. *Bull. Chem. Soc. Jpn.* **1958**, *31* (9), 1081–1082. DOI: 10.1246/bcsj.31.1081.

(2) Vochten, R.; Petre, G. Study of the heat of reversible adsorption at the air-solution interface. II. Experimental determination of the heat of reversible adsorption of some alcohols. *J. Colloid Interface Sci.* **1973**, *42* (2), 320–327. DOI: 10.1016/0021-9797(73)90295-6.

(3) Góral, M.; Wiśniewska-Gocłowska, B.; Mączyński, A. Recommended Liquid–Liquid Equilibrium Data. Part 4. 1-Alkanol–Water Systems. *J. Phys. Chem. Ref. Data* **2006**, *35* (3), 1391–1414. DOI: 10.1063/1.2203354.

(4) Stephenson, R.; Stuart, J.; Tabak, M. Mutual solubility of water and aliphatic alcohols. *J. Chem. Eng. Data* **1984**, *29* (3), 287–290. DOI: 10.1021/je00037a019.

(5) Sangster, J. Octanol-Water Partition Coefficients of Simple Organic Compounds. *J. Phys. Chem. Ref. Data* **1989**, *18* (3), 1111–1229. DOI: 10.1063/1.555833.

(6) OECD. *Partition Coefficient (n-octanol/water): HPLC Method*, 2014.

(7) Lorentz, H. A. Über die Anwendung des Satzes vom Virial in der kinetischen Theorie der Gase. *Ann. Phys.* **1881**, *248* (1), 127–136. DOI: 10.1002/andp.18812480110.

(8) Wolbach, J. P.; Sandler, S. I. Using Molecular Orbital Calculations To Describe the Phase Behavior of Cross-associating Mixtures. *Ind. Eng. Chem. Res.* **1998**, *37* (8), 2917–2928. DOI: 10.1021/ie970781l.

(9) Floriano, W. B.; Nascimento, M. A. C. Dielectric constant and density of water as a function of pressure at constant temperature. *Braz. J. Phys.* **2004**, *34* (1), 38–41. DOI: 10.1590/S0103-97332004000100006.

(10) Ascani, M.; Held, C. Prediction of salting-out in liquid-liquid two-phase systems with ePC-SAFT: Effect of the Born term and of a concentration-dependent dielectric constant. *Z. Anorg. Allg. Chem.* **2021**, *647* (12), 1305–1314. DOI: 10.1002/zaac.202100032.

(11) Andeen, C.; Fontanella, J.; Schuele, D. Low-Frequency Dielectric Constant of LiF, NaF, NaCl, NaBr, KCl, and KBr by the Method of Substitution. *Phys. Rev. B* **1970**, *2* (12), 5068–5073. DOI: 10.1103/PhysRevB.2.5068.

(12) Fritschka, E.; Sadowski, G. Rigorous modeling the pH-dependent solubility of weak acids, weak bases and their salts. *Fluid Ph. Equilib.* **2024**, *580*, 114039. DOI: 10.1016/j.fluid.2024.114039.

(13) Cameretti, L. F.; Sadowski, G. Modeling of aqueous amino acid and polypeptide solutions with PC-SAFT. *Chem. Eng. Process. Process Intensif.* **2008**, *47* (6), 1018–1025. DOI: 10.1016/j.cep.2007.02.034.

(14) Gross, J.; Sadowski, G. Application of the Perturbed-Chain SAFT Equation of State to Associating Systems. *Ind. Eng. Chem. Res.* **2002**, *41* (22), 5510–5515. DOI: 10.1021/ie010954d.

(15) Prudic, A.; Kleetz, T.; Korf, M.; Ji, Y.; Sadowski, G. Influence of copolymer composition on the phase behavior of solid dispersions. *Mol. Pharm.* **2014**, *11* (11), 4189–4198. DOI: 10.1021/mp500412d. Published Online: Oct. 21, 2014.

(16) Ruether, F.; Sadowski, G. Modeling the solubility of pharmaceuticals in pure solvents and solvent mixtures for drug process design. *J. Pharm. Sci.* **2009**, *98* (11), 4205–4215. DOI: 10.1002/jps.21725.

(17) Cassens, J.; Prudic, A.; Ruether, F.; Sadowski, G. Solubility of Pharmaceuticals and Their Salts As a Function of pH. *Ind. Eng. Chem. Res.* **2013**, *52* (7), 2721–2731. DOI: 10.1021/ie302064h.

(18) Paus, R.; Ji, Y.; Vahle, L.; Sadowski, G. Predicting the Solubility Advantage of Amorphous Pharmaceuticals: A Novel Thermodynamic Approach. *Mol. Pharm.* **2015**, *12* (8), 2823–2833. DOI: 10.1021/mp500824d. Published Online: Jul. 8, 2015.

(19) Held, C.; Reschke, T.; Mohammad, S.; Luza, A.; Sadowski, G. ePC-SAFT revised. *Chem. Eng. Res. Des.* **2014**, *92* (12), 2884–2897. DOI: 10.1016/j.cherd.2014.05.017.

(20) Lehmkemper, K.; Kyeremateng, S. O.; Heinzerling, O.; Degenhardt, M.; Sadowski, G. Long-Term Physical Stability of PVP- and PVPVA-Amorphous Solid Dispersions. *Mol. Pharm.* **2017**, *14* (1), 157–171. DOI: 10.1021/acs.molpharmaceut.6b00763. Published Online: Dec. 7, 2016.

(21) Lübbert, C.; Sadowski, G. Moisture-induced phase separation and recrystallization in amorphous solid dispersions. *Int. J. Pharm.* **2017**, *532* (1), 635–646. DOI: 10.1016/j.ijpharm.2017.08.121. Published Online: Sep. 1, 2017.

(22) Veith, H.; Voges, M.; Held, C.; Albert, J. Measuring and Predicting the Extraction Behavior of Biogenic Formic Acid in Biphasic Aqueous/Organic Reaction Mixtures. *ACS omega* **2017**, *2* (12), 8982–8989. DOI: 10.1021/acsomega.7b01588. Published Online: Dec. 14, 2017.

(23) Ding, Y.; Cui, W.; Zhang, Z.; Ma, Y.; Ding, C.; Lin, Y.; Xu, Z. Solubility and Pharmacokinetic Profile Improvement of Griseofulvin through Supercritical Carbon Dioxide-Assisted Complexation with HP-γ-Cyclodextrin. *Molecules* **2023**, *28* (21). DOI: 10.3390/molecules28217360. Published Online: Oct. 31, 2023.

(24) Miwa, Y.; Hamamoto, H.; Ishida, T. Lidocaine self-sacrificially improves the skin permeation of the acidic and poorly water-soluble drug etodolac via its transformation into an ionic liquid. *Eur. J. Pharm. Biopharm.* **2016**, *102*, 92–100. DOI: 10.1016/j.ejpb.2016.03.003. Published Online: Mar. 2, 2016.

(25) Mohammadzade, M.; Barzegar-Jalali, M.; Jouyban, A. Solubility of naproxen in 2-propanol+water mixtures at various temperatures. *J. Mol. Liq.* **2015**, *206*, 110–113. DOI: 10.1016/j.molliq.2015.01.052.

(26) Garzn, L. C.; Martnez, F. Temperature Dependence of Solubility for Ibuprofen in Some Organic and Aqueous Solvents. *J. Solution Chem.* **2004**, *33* (11), 1379–1395. DOI: 10.1007/s10953-004-1051-2.
